# Decellularized vascular matrix material–based TEVG coated with PRP for anti-degradation and anti-inflammation

**DOI:** 10.1097/JS9.0000000000003521

**Published:** 2025-09-24

**Authors:** Shui-Lan Wu, Jian-Yi Xu, Xu-Heng Sun, Hong-Jing Jiang, Rong-Hua Yang, Zhan-Yi Lin

**Affiliations:** a Department of Burn and Plastic Surgery, Guangzhou First People’s Hospital, Guangzhou, Guangdong, China; b Ji Hua Laboratory, Ji Hua Institute of Biomedical Engineering Technology, Foshan, Guangdong, China; c Department of Cardiology, Guangdong Provincial People’s Hospital (Guangdong Academy of Medical Sciences), South Medical University, Guangzhou, Guangdong, China

**Keywords:** macrophages, PRP, smooth muscle cells, tissue-engineered vessels

## Abstract

**Background:**

Vascular regeneration is closely associated with the inflammatory response and the degradation rate of implants. Platelet-rich plasma (PRP) contains various cytokines and proteins, and autologous PRP can be used to treat implants to reduce the inflammatory response.

**Objective:**

To reduce the immune rejection response and degradation rate of implants *in vivo* by incorporating different derivatives of PRP.

**Methods:**

Tissue-engineered vascular grafts (TEVGs) were separately mixed with phosphate-buffered saline (PBS), fibrin (FIB), platelet growth factor (PGF), and a PGF blend to prepare different extracellular matrix (ECM) implants for cell co-culture and subcutaneous transplantation experiments in rats. The cytokines transforming growth factor-beta, interleukin-10, and transforming growth factor-alpha released by macrophages were detected by enzyme-linked immunosorbent assay. Tissue morphology was examined using hematoxylin and eosin (H&E staining), Masson’s trichrome staining, and scanning electron microscopy. The impact of TEVGs on macrophages was evaluated through immunofluorescence and Western blotting. Subcutaneous transplantation in rats was assessed using H&E and Masson’s staining, along with immunofluorescence staining for CD206 and CD86 to observe cell numbers and the M2/M1 (alternatively activated macrophages/classically activated macrophages) ratio.

**Results:**

PBS, FIB, PGF, and the PGF blend exhibited distinct morphologies under scanning electron microscopy. Both *in vitro* and *in vivo* studies demonstrated an increase in the M2/M1 ratio with PGF and PGF-coated groups, improved water absorption capacity, and delayed metabolism of ECM materials *in vivo*. Additionally, PRP downregulated multiple inflammation-related genes, thereby reducing the inflammatory response.

**Conclusion:**

PGF and PGF-coated TEVGs reduced the immune rejection response in subcutaneous transplantation and decreased degradation by mitigating collagen loss in the implants.

**HIGHLIGHTS**

- The primary objective of this study was to investigate the potential of platelet-rich plasma (PRP) to promote and support vascular grafting.
- Following the addition of PRP, the graft materials exhibited M2 macrophage polarization both *in vivo* and *in vitro*.
- PRP was found to prolong the degradation time of the graft material *in vivo*, thereby providing an extended time window for subsequent recellularization processes.

## Introduction

Tissue-engineered blood vessels (TEBVs) offer promising solutions to address the clinical shortage of small-caliber vascular grafts. A team led by Laura utilized polyglycolic acid (PGA) scaffolds and smooth muscle cells (SMCs) for co-culture over 8 weeks to obtain vascular matrix materials, which demonstrated success in animal experiments and have progressed to clinical trials^[1]^. Compared to the clinically applied polymeric vascular graft material polytetrafluoroethylene (PTFE), these cell-derived extracellular matrix (ECM) materials exhibit superior regenerative potential and reduced infection risk^[2]^. In a rat model, subcutaneous transplantation of decellularized vascular matrix material–based tissue-engineered vascular grafts (TEVGs), obtained by decellularizing TEBVs, revealed significant morphological changes after 3 weeks of implantation. Notably, extensive cellular infiltration – primarily inflammatory cells – was observed as early as the first week, which was closely associated with material degradation and tissue metabolism^[3]^. Material degradation and the inflammatory response remain critical challenges in vascular tissue engineering. To mitigate the inflammatory response during vascular graft implantation and enhance the *in vivo* regenerative potential, previous studies have shown that TEVGs, by removing cellular components, can substantially reduce immune rejection^[4]^. Additionally, TEVG materials mimic the ECM structure of native blood vessels and exhibit superior regenerative plasticity. Although TEVGs can eliminate a significant portion of immunogenic components through decellularization, excessive removal of cellular remnants during TEVG fabrication may lead to protein loss. Furthermore, residual PGA in TEVG materials can provoke inflammatory reactions *in vivo*^[5]^. Previous efforts to remove immunogenic substances have rendered TEVGs structurally fragile. Reducing the immune rejection response to vascular scaffolds and extending their *in vivo* degradation period have become major challenges in vascular transplantation. Studies have demonstrated that early infiltrating cells in vascular scaffolds are primarily inflammatory cells, which are closely associated with tissue degradation and metabolic activity. To mitigate the inflammatory response during vascular graft implantation and enhance *in vivo* regenerative capacity, some researchers have employed immunosuppressants such as cyclosporine and methylprednisolone to suppress the host immune response, thereby reducing post-transplant inflammation^[6]^. Alternatively, regulation of the expression and release of inflammation-related cytokines – such as tumor necrosis factor (TNF), interleukin (IL)-1, and IL-6 – may also reduce inflammation after transplantation. Using natural ECM as a vascular graft material is preferable, as it possesses inherent biocompatibility and bioactivity, and may reduce immune rejection and inflammation^[7]^. However, these approaches are complex and costly, limiting their widespread clinical applicability. Whether an effective and efficient strategy exists to regulate the graft–host interaction and thereby reduce transplant-related immune responses remains to be determined.

Platelet-rich plasma (PRP) primarily contains fibrinogen, fibronectin, growth factors, cytokines, and other bioactive molecules. These components play critical roles in stimulating cell proliferation, promoting angiogenesis, and attenuating inflammatory processes – without substantially increasing the economic burden on patients. To date, PRP has been widely applied in tissue engineering fields such as cartilage regeneration^[8]^, bone tissue engineering^[9]^, skin repair^[10]^, and neural tissue engineering^[11]^. In recent years, the application of PRP in vascular tissue engineering has also gained attention. For example, our recent study demonstrated that PRP enhances the adhesion and migratory capacity of SMCs on PGA scaffolds, thereby promoting collagen production during the later stages of tissue formation^[12]^. PRP can be extracted from the patient’s own blood or from compatible donors, effectively addressing the issue of material biocompatibility during TEVG application. However, the impact of PRP on subcutaneous transplantation of ECM-treated TEVGs has not yet been fully explored. The long-term outcomes of vascular transplantation are strongly associated with the inflammatory response and the degradation rate of the implanted material^[13]^. Strategies to reduce the degradation rate of vascular grafts and provide a sufficient time window for subsequent vascular cell ingrowth include surface modification techniques. For instance, Yilgor *et al* designed a drug delivery system with controlled release functionality – such as the bone morphogenetic protein (BMP)-2/BMP-7 system – that releases anti-inflammatory, antibacterial, or pro-angiogenic agents to exert anti-inflammatory effects on transplants^[14,15]^. However, such systems are complex and present challenges for clinical scalability. Studies have shown that PRP can reduce the production of inflammatory cytokines, such as IL-17A and IL-1β, thereby mitigating the inflammatory response – primarily in the context of skin repair and regeneration^[16]^. Whether PRP modulates the inflammatory response to TEVGs *in vivo* and whether it improves the degradation and metabolic profile of vascular scaffold materials remains unknown.

After decellularizing TEBVs, we obtained TEVGs, which were subsequently freeze-dried at low temperatures. The TEVGs were then treated with phosphate-buffered saline (PBS), fibrin (FIB), platelet growth factor (PGF; derived from PRP), and PGF^−^ (PGF without FIB; derived from PGF), respectively. Following enzymatic digestion, the materials were evaluated for macrophage polarization *in vitro*. In addition, the PBS-, FIB-, PGF-, and PGF^−^-treated materials were subcutaneously transplanted into rats. Samples were collected at 1, 2, and 3 weeks, and immunofluorescence staining for CD206, CD86, and DAPI was performed to assess macrophage polarization (M2/M1 [alternatively activated macrophages/classically activated macrophages] phenotype). Hematoxylin and eosin (H&E) staining was used to quantify inflammatory cell infiltration, and collagen content at each time point was measured using Masson’s trichrome staining. The hydroxyproline assay was conducted to quantify collagen deposition using samples collected at each time point. Finally, transcriptomic analysis was performed to investigate the potential mechanisms of PRP action during subcutaneous transplantation. This study adhered to the 2025 TITAN guidelines throughout its design, experimentation, and manuscript preparation stages, and did not involve any improper use of AI tools^[17–19]^.

## Materials and methods

This study was conducted in accordance with the ARRIVE (Animal Research: Reporting of *In Vivo* Experiments) guidelines^[20]^. All Sprague–Dawley (SD) rats were purchased from the local experimental animal center. A total of 48 male rats, aged 4–6 weeks and weighing 100–150 g, were used for subcutaneous transplantation experiments.

Nonwoven PGA material was procured from Biomedical Structures (Warwick, RIUSA). SMCs were isolated from bovine aortic tissue obtained from a local abattoir.

### Acquisition, decellularization, and dissolution of TEBV to form TEVG

TEBVs were generated by dynamic cultivation of bovine SMCs on nonwoven PGA scaffolds using a peristaltic pump over an 8-week period. The decellularization protocol was adapted from the method established by Pellegata and colleagues^[5]^. The resulting decellularized TEBVs – referred to as TEVGs – were freeze-dried and stored at −80 °C until further use. DNA content was measured using a DNA extraction kit (Thermo Fisher Scientific, USA) according to the manufacturer’s protocol.

Preparation of the TEVG solution was based on previously published methods^[21]^. In brief, TEVGs were digested into solution by adding pepsin at a concentration of 10 mg/mL (w/v). The digestion was performed under shaking conditions at 37 °C. Once fully solubilized, the solution was neutralized using 0.1 N sodium hydroxide (NaOH; 1:10 volume) and 10× PBS (1:9 volume) to achieve a final pH of 7.4. The neutralized solution was then stored at 4 °C for subsequent use.

### Preparation of PRP

PRP was prepared using a conventional two-step centrifugation method. Blood was initially collected from rats in anticoagulant tubes containing sodium citrate (KWS, China). Two-step centrifugation was then performed at 300 × g for 15 minutes, followed by 600 × g for 10 minutes, to obtain a high-yield PRP fraction. Platelet counts were conducted for both whole blood and PRP using an optical microscope to ensure that the platelet concentration in PRP was approximately five times higher than that in whole blood^[22]^. Platelets were stained and analyzed using the Richter staining method (Biosharp, China). Subsequently, PRP underwent repeated freeze–thaw cycles, followed by centrifugation to obtain a cell-free PRP solution. One portion of the solution was used directly for subsequent experiments (PGF), while another was subjected to calcium ion activation to remove FIB components (PGF^−^). The remaining PRP was stored at −80 °C for later use.

### Scanning electron microscopy

Samples from the PBS (TEVG treated with PBS), FIB (TEVG treated with 2 g/mL fibrinogen), PGF (TEVG treated with PRP containing fibrinogen precursors), and PGF^−^ (TEVG treated with PRP lacking fibrinogen precursors) groups were fixed in 3 mL of 2.5% glutaraldehyde. The samples were rinsed with 0.1 mol/L sodium dimethylaminomethylphosphonate buffer (pH 7.4) and stored in buffer solution at 4 °C overnight. After 24 hours, the TEVGs from each group were immersed in 1% citric acid for 1 hour and then repeatedly washed with buffer solution^[23]^. Samples were then observed using a scanning electron microscope (S-3500N, Japan).

### Growth factor release experiment and cytokine detection

To determine the release profile of growth factors from PRP-treated TEVG materials, platelet-derived growth factor-BB (PDGF-BB) and vascular endothelial growth factor (VEGF) were used as assay markers. TEVG samples weighing 10 μg (dry weight) were soaked in PGF and PGF^−^ 24-hour solutions and then placed in 96-well plates (Corning, USA) for incubation. A volume of 200 μL of TEVG solution was added to each well. Culture media were collected at various time points (2, 4, 6, 8, 10, 12, and 14 days), stored at −80 °C, and later analyzed using enzyme-linked immunosorbent assay (ELISA; DuoSet®, USA) according to the manufacturer’s protocol to quantify the concentrations of PDGF-BB and VEGF released from the materials.

To assess cytokine secretion by macrophages, cells were seeded at a density of 5 × 10^4^ cells per well in six-well plates. After culturing for 7 days, the medium was removed and replaced with RPMI-1640 medium for an additional 24-hour incubation. The culture supernatant was then collected, and levels of transforming growth factor-beta (TGF-β), IL-10, and TGF-alpha (TGF-α) were measured using an ELISA kit (NEOBIOSCIENCE, China) in accordance with the manufacturer’s instructions.

### Material water absorption test

Water absorption was defined as the percentage increase in material mass due to water uptake, relative to its dry mass. After treatment, samples from different experimental groups were freeze-dried, and their dry weight (M) was recorded. These samples were then immersed in double-distilled water (ddH_2_O), and the saturated weight (m) was measured. The water absorption rate was calculated using the following formula: Water absorption (%) = (m − M)/M.

### isolation and identification of SMCs

For the isolation of SMCs, approximately 5 cm segments of bovine aorta were obtained from the Nan-Hai Abattoir in Foshan, Guangdong Province. The medial layer of the aorta, which is notably thicker than that of human aortas, was selected for subsequent experiments. This layer, taken from the mid-section of the vessel whenever possible, was cut into small fragments approximately 0.2 cm × 0.2 cm in size. Primary cultures of SMCs were established using a tissue adhesion method. The vessel fragments were evenly distributed onto T25 culture flasks (Corning, USA) and incubated at 37 °C in a humidified atmosphere with 5% CO_2_ (Thermo, USA). Dulbecco’s Modified Eagle Medium/Nutrient Mixture F-12 (Corning, USA) supplemented with 20% fetal bovine serum (FBS; Gibco, USA) was used. After 4 hours, the flasks were left undisturbed for 1 week to prevent tissue detachment and promote cell outgrowth.

After 2 weeks, cells were passaged up to passage 3. Approximately 5 × 10^4^ cells were collected using a cell scraper (Biosharp, China) and subjected to immunofluorescence staining according to the manufacturer’s protocol. The cells were incubated with primary antibodies against calponin and α-smooth muscle actin (α-SMA) (1:500 dilution; Abcam, USA), followed by DAPI nuclear staining (Solarbio, China) for visualization^[24]^.

### CCK-8 assay

To assess cell viability, SMCs were seeded in 96-well plates at a density of 4000 cells per well. Cells were then treated with TEVG solutions at concentrations of 50, 100, 150, 200, and 250 ng/mL for 24 hours. According to the manufacturer’s instructions (Dojindo, Japan), 10% CCK-8 reagent was added to each well, and the plates were incubated at 37 °C for 1.5 hours. The absorbance at 450 nm was measured using a microplate reader, and results were compared across groups.

### Extracorporeal isolation and cultivation of macrophages

Macrophages were isolated from the femurs of healthy 6-week-old male SD rats^[25]^. Bone marrow was flushed from the femurs using PBS. After settling, the bottom layer containing bone fragments was removed, and the remaining cell suspension was treated with a 0.3% sodium chloride solution to lyse red blood cells. Following centrifugation, the cell pellets were resuspended in RPMI-1640 medium (Gibco, USA) supplemented with 10% FBS and cultured in suspension to promote macrophage differentiation. After 1 week of culture, macrophages were treated with 20 ng/mL macrophage colony-stimulating factor (M-CSF) for 24 hours. Subsequently, both macrophages and SMCs were treated with one of the following solutions for 24 hours: PBS (TEVG solution at 200 ng/mL), FIB (TEVG solution containing 2 g/mL fibrinogen), PGF (TEVG solution containing fibrinogen precursors), or PGF^−^ (TEVG solution without fibrinogen precursors), prior to immunofluorescence and Western blot (WB) analyses.

### Cell immunofluorescence and flow cytometry:

Cells were fixed with 4% paraformaldehyde for 10 minutes, then permeabilized with 0.1% Triton X-100 in PBS. After blocking with 1% bovine serum albumin in PBS for 30 minutes, cells were incubated overnight at 4 °C with primary antibodies: calponin (CNN, 1:300; Abcam, USA), α-SMA (1:300; Abcam, USA), CD206 (1:250; Abcam, USA), and inducible nitric oxide synthase (iNOS, 1:250; Abcam, USA). The following day, cells were washed three times with PBST while protected from light. Fluorescent secondary antibodies were applied at a tenfold dilution relative to the corresponding primary antibodies. Nuclei were counterstained with anti-fade mounting medium containing DAPI (Merck, China) for 1 minute, and samples were imaged using a confocal microscope (Thermo Fisher Scientific, USA).

For flow cytometry analysis, cells from each group were harvested, washed twice with cold PBS, and resuspended in staining buffer (PBS containing 1% FBS). Cells were then incubated with CD163-APC and CD206-FITC (eBioscience, USA) for 90 minutes at 4 °C in the dark. After staining, cells were washed and analyzed using a flow cytometer (BD FACSCanto II, USA). Data were processed using FlowJo software (version 10.8.1).

### WB analysis

Macrophages and previously cultured SMCs from each experimental group were harvested for protein extraction. Proteins were isolated using a protein extraction kit containing protease inhibitors and radioimmunoprecipitation assay buffer (Solarbio, China). Total protein concentration was measured using a bicinchoninic acid assay kit (Biosharp, China), following the manufacturer’s protocol. The following primary antibodies were used: anti-calponin (CNN, 1:500; Abcam, USA), anti-α-α-SMA (1:2000; Abcam, USA), anti-CD206 (1:1000; Abcam, USA), anti-iNOS (1:500; Abcam, USA), anti-β-tubulin (1:15 000; Abcam, USA), and anti-glyceraldehyde 3-phosphate dehydrogenase (GAPDH, 1:10 000; Abcam, USA). Protein detection was performed using enhanced chemiluminescence substrate (Pierce) on polyvinylidene fluoride membranes. The membranes were imaged using a digital exposure system (Tanon, Shanghai, China). WB bands were analyzed using ImageJ software. Band intensities were quantified by calculating grayscale values, and target protein expression was normalized to the corresponding internal control band to reduce experimental variability.

### Subcutaneous implantation in rats

Decellularized TEVGs were divided into four experimental groups – PBS, FIB, PGF, and PGF^−^ – based on the study design. TEVGs from each group were evenly cut into pieces measuring approximately 1 cm × 1 cm. These fragments were then subcutaneously implanted into rats. A total of 48 rats were randomly assigned to four groups (*n* = 12 per group). Tissue samples were harvested at 1, 2, and 3 weeks post-implantation. Data from week 4 were excluded due to advanced degradation and resorption of the grafts, which made representative histological analysis unfeasible. All animals were housed under specific pathogen-free conditions with ambient temperature maintained at 20–22 °C, relative humidity between 40% and 60%, and a 12-hour light/dark cycle to mimic natural circadian rhythms. Anesthesia was administered using 0.2% isoflurane via inhalation. Implanted materials were retrieved biweekly for subsequent analyses.

### H&E and Masson’s trichrome staining

After tissue collection, the samples were gently rinsed three times with PBS and fixed in 10% formaldehyde solution. Fixed tissues were again washed with PBS, embedded in paraffin, and sectioned. The sections were stained using H&E and Masson’s trichrome protocols for histological analysis.

### Tissue immunofluorescence staining

After paraffin tissue sections were prepared, immunofluorescence staining was performed. Sections were fixed overnight in 4% paraformaldehyde at 4 °C and subsequently rinsed with PBS. The sections were then dehydrated using a graded ethanol series and treated with butanol for 1 hour. Paraffin embedding was conducted by incubating the samples in a 65 °C dry oven for 3 hours, followed by microtome sectioning. Subsequent steps followed the procedure described for cell immunofluorescence staining. The primary antibodies used – CD206 and iNOS – were both diluted at 1:200 (Abcam, USA). Image quantification was performed using CaseViewer software by randomly selecting five fields (magnification 40×) surrounding the graft area. Each field was defined by a circular area with a 100-μm diameter. The number of positive cells per field was counted, and the average was calculated for statistical analysis.

### Hydroxyproline quantification

Tissue collagen content was assessed using the Hydroxyproline Detection Kit (Solarbio, China). Samples were weighed and labeled according to the manufacturer’s instructions. The tissues were finely minced to facilitate digestion, then mixed with 2 mL of hydrochloric acid-containing extraction solution. Samples were incubated at 110 °C for 4–6 hours. After cooling, the pH was adjusted to 6.8–7.4 using NaOH solution. The samples were then centrifuged at 16 000 rpm for 20 minutes at 25 °C. The resulting supernatant was collected for analysis. The spectrophotometer was preheated for 30 minutes, and the absorbance was measured at 560 nm after zeroing with distilled water. A standard curve was generated using known concentrations, and the hydroxyproline content was calculated based on the measured absorbance values^[26]^.

### RNA extraction and quantitative real-time polymerase chain reaction

After 1 week of subcutaneous transplantation, samples from both the PRP-treated experimental group (PGF) and the untreated control group (without PRP) were subjected to transcriptome analysis (*n* = 5 per group). Total RNA was extracted using TRIzol reagent (Thermo Fisher Scientific, USA) according to the manufacturer’s protocol. The extracted RNA was then purified and concentrated using ethanol precipitation. RNA integrity was assessed using the Bioanalyzer 2100 (Agilent Technologies, USA).

Purified RNA was reverse-transcribed into complementary DNA and labeled for high-throughput sequencing. Libraries were prepared using the SMART method and sequenced on the Illumina NovaSeq 6000 platform. Sequencing reads were aligned using HISAT, assembled, and quantified with StringTie. Differential gene expression was analyzed using edgeR and visualized in R software through heatmaps, volcano plots, and principal component analysis^[27]^.

Quantitative real-time polymerase chain reaction (RT-qPCR) was conducted using the qTOWER^3^ G real-time PCR system (Analytik Jena, Germany), following the manufacturer’s instructions (KeyGEN, China). The primers used for amplification are listed in Table 1. All reactions were performed in triplicate, and relative gene expression was calculated using the 2^−ΔΔCt^ method with GAPDH as the internal control.

**Table 1:** Primer sequences for RT-qPCR

Gene	Primer sequences
CFB	F:5′-GAGCGCAACTCCAGTGCTT-3′
	R:5′-GGAGCCGCCTTTGATCTCTAC-3′
CXCL6	F:5′-GTTCCATCTCGCCATTCATGC-3′
	R:5′-GCGGCTATGACTGAGGAAGG-3′
Il12rb2	F:5′-TGCCCCTGTGATTCCTCTTG-3′
	R:5′-ACTGGGATAATGTGAACAGCCT-3′
Il17a	F:5′-TCAGCGTGTCCAAACACTGAG-3′
	R:5′-CGCCAAGGGAGTTAAAGACTT-3′
MASP1	F:5′-ACCGTGGAGCTAAACGAAATG-3′
	R:5′-TCCAAGTTGAAGTGCATGAAGT-3′
MAPK10	F:5′-AGGTGGACAACCAGTTCTACA-3′
	R:5′-GCACAGACTATTCCCTGAGCC-3′
Coagulation factor II	F:5′-CCGAAAGGGCAACCTAGAGC-3′
	R:5′-GGCCCAGAACACGTCTGTG-3′
Coagulation factor IX	F:5′-ATGCTGGTGCCAAGTTGGATT-3′
	R:5′-CTCAGTGCAGGAACAAATTACCT-3′
GAPDH	F:5′-GGCGTGAACCACGAGAAGTATAA-3′
	R:5′-CCCTCCACGATGCCAAAGT-3′

### Statistical analysis

All quantitative data were reported as mean ± standard deviation with *n* = 5 per group. Assuming equal variances, statistical significance between groups was evaluated using a two-sample *t*-test. A *P*-value <0.05 was considered statistically significant.

## Results

### Materials characterization and cell identification

**Figure 1.**
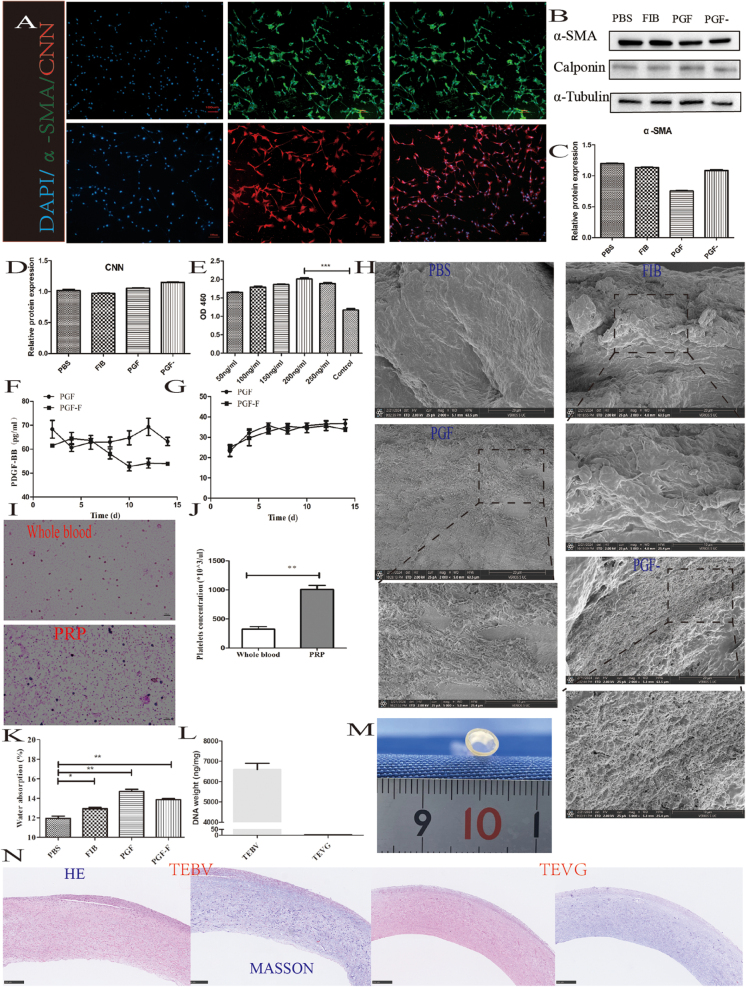
Materials characterization and cell identification. (A) Immunofluorescence staining images for phenotypic identification of smooth muscle cells; (B) expression profiles of phenotypic proteins in different experimental groups; (C) semi-quantitative analysis of α-SMA protein expression from WB images.; (D) semi-quantitative analysis of CNN protein expression from WB images; (E) effect of TEVG solution at varying concentrations on smooth muscle cell proliferation; (F) release profile of PDGF-BB; (G) profile of VEGF; (H) SEM images of TEVGs treated with different groups; (I) Wright’s staining of whole blood and PRP; (J) platelet count comparison between whole blood and PRP in rats; (K) water absorption capacity of materials across experimental groups; (L) DNA quantification in TEBV and TEVG; (M) Gross morphology of TEBV; and (N) hematoxylin and eosin (HE) and Masson’s trichrome staining of TEBV and TEVG. Scale bars: 10 µm, 20 µm, and 100 µm. Statistical significance: *P < 0.05, **P < 0.01, ***P < 0.001.

Immunofluorescence staining confirmed that the SMCs used in this study expressed the smooth muscle-specific markers CNN and α-SMA (Fig. 1A). Consistent protein expression of CNN and α-SMA was also observed in WB analysis. Semi-quantitative analysis of WB band intensities is shown in Figure 1B–D, revealing no significant differences between the experimental groups. Figure 1E illustrates the effects of TEVG solution, applied at varying concentrations, on SMCs. CCK-8 assay results on day 3 indicated that TEVG solutions at different concentrations exhibited no cytotoxicity toward SMCs. The cell proliferation assay showed that from a concentration of 50 ng/mL onward, SMCs demonstrated increased proliferative capacity. However, this effect reached statistical significance only at 200 ng/mL, which was therefore selected for use in subsequent experiments.

Using a rat-specific growth factor detection kit, sustained release profiles of VEGF and PDGF-BB were observed in both the PGF and PGF^−^ groups over a 14-day period. VEGF levels remained stable in both groups throughout the observation period. However, a decline in PDGF-BB levels was observed in the PGF^−^ group on day 10, possibly due to the absence of fibrinogen. Natural plasma proteins are known to exert sustained-release effects on growth factors; thus, removal of specific proteins such as fibrinogen may alter release kinetics (Fig. 1F, G). Whole blood was collected from rat hearts using anticoagulant-treated tubes, yielding bright red blood. Wright’s staining revealed sparse light purple-stained platelet areas in whole blood (Fig. 1I, upper panel), whereas PRP exhibited numerous positively stained regions (Fig. 1I, lower panel). Quantitative analysis showed that the platelet count in PRP was 4.10 ± 0.13 times higher than that in whole blood, aligning with established PRP application standards (Fig. 1J).

Scanning electron microscopy (SEM) revealed an overall smooth surface on TEVGs treated with PBS alone, with occasional creases and depressions observed (Fig. 1H, PBS). TEVGs treated with FIB exhibited filamentous FIB fibers throughout the surface layer, in contrast to the untreated group, which appeared relatively loose – possibly due to differences in soaking concentration. PGF-treated TEVGs showed the presence of filamentous proteins across the longitudinal section, while PGF^−^-treated TEVGs (in which fibrinogen was removed) displayed fewer proteins compared to the PGF group.

Water absorption rate testing across the four experimental groups demonstrated significantly increased absorption compared to PBS alone, with statistically significant differences observed among the treatment groups. The PGF group exhibited the highest water absorption rate, followed by the PGF^−^ and FIB groups, while the PBS group showed the lowest rate. This trend was consistent with protein content observed via SEM – higher protein content corresponded to increased water absorption (Fig. 1K).

Figure 1M presents an overview of TEBV, while Figure 1N shows the results of H&E and Masson’s trichrome staining of TEBV and TEVG. The staining did not reveal complete cellular structures, and the scaffold structure of TEVG remained largely intact. As shown in Figure 1L, the DNA content of TEVG (3.73 ± 0.74 ng/mg) was significantly lower than that of TEBV (6590.67 ± 530.61 ng/mg) and well below the accepted safety threshold of 50 ng/mg.

### Effect of TEVG of different groups on macrophage phenotype, light microscopy, and WB experiments

**Figure 2.**
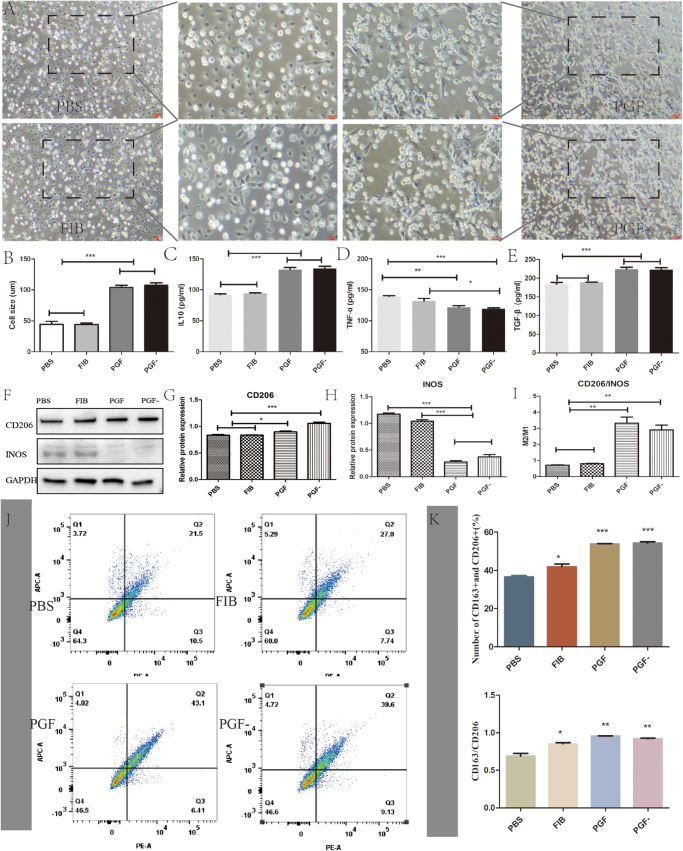
The effect of PRP on macrophage polarization. (A) Light microscopy images of macrophages treated for 24 hours with PBS, FIB, PGF, and PGF^−^; (B) measurement of macrophage length; (C) quantification of IL-10 expression; (D) quantification of TNF-α expression; (E) quantification of TGF-β expression; (F) Western blot images showing expression of CD206 and INOS across groups; (G) densitometric analysis of CD206 expression; (H) densitometric analysis of INOS expression; (I) ratio of CD206 to INOS expression in different experimental groups; (J) flow cytometry images showing expression of CD163 and CD206; and (K) quantitative analysis of CD163⁺ and CD206⁺ cell populations from flow cytometry. Scale bar: 100 μm; *P < 0.05, **P < 0.01, ***P < 0.001.

Observation of macrophage morphology under light microscopy – along with magnified insets in the upper-right corners – revealed that the PGF and PGF^−^ experimental groups predominantly exhibited elongated cell shapes (Fig. 2A). Measurement of the average macrophage length across five distinct fields of view, followed by statistical analysis, indicated significantly longer cell lengths in the PGF and PGF^−^ groups compared to the PBS and FIB groups (Fig. 2B). Growth factor analysis showed that levels of TGF-α, secreted by M1 macrophages, were higher in the PBS and FIB groups, whereas IL-10 and TGF-β – secreted by M2 macrophages – were more elevated in the PGF and PGF^−^ groups (Fig. 2C–E).

WB was performed to evaluate the influence of ECM components on macrophage polarization *in vitro*. Results showed higher expression of the M1 marker INOS in the PBS and FIB groups compared to the PGF and PGF^−^ groups, with statistically significant differences. Conversely, the expression of the M2 marker CD206 was highest in the PGF^−^ and PGF groups. The CD206/INOS ratio followed the order: PGF > PGF^−^ > PBS/FIB (Fig. 2F–I), suggesting that the presence of fibrous proteins alone has minimal influence on M2/M1 polarization of macrophages. Flow cytometry analysis revealed a higher total number of CD163^+^ and CD206^+^ cells in the PGF and PGF^−^ treatment groups (PBS: 36.6 ± 1.23; FIB: 41.93 ± 2.60; PGF: 53.86 ± 0.25; PGF^−^: 54.33 ± 1.10) compared to the control group. Furthermore, the CD163/CD206 ratio was elevated in the PGF and PGF^−^ groups relative to the control (Fig. 2J, K).

### Effects of TEVG of different experimental groups on macrophage phenotypes: cell immunofluorescence staining

**Figure 3.**
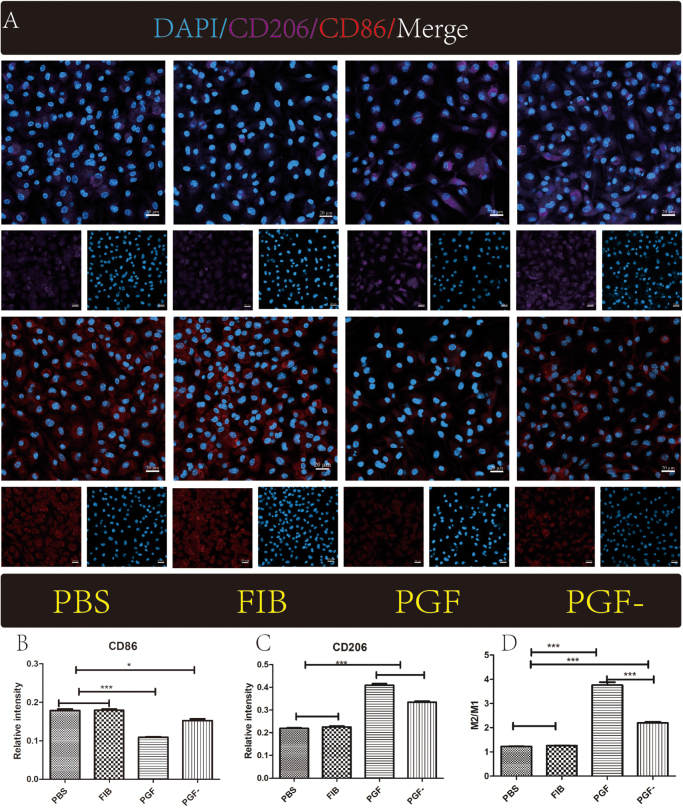
The effect of PRP on macrophage polarization. (A) Immunofluorescence images showing the interaction between TEVG materials and macrophages; (B) relative expression of CD86 (M1 macrophage marker); (C) relative expression of CD206 (M2 macrophage marker); and (D) ratio of CD206 to CD86 (M2/M1) in different experimental groups. Scale bar: 100 μm; *P < 0.05, ***P < 0.001.

To evaluate macrophage polarization, immunofluorescence staining was used to detect the expression of CD86 (a marker for M1 macrophages) and CD206 (a marker for M2 macrophages) following stimulation with different TEVG experimental groups. Fluorescence signals for both M1 and M2 macrophages were detected in all four groups. Notably, the PGF and PGF^−^ groups exhibited stronger M2-associated fluorescence and weaker M1-associated fluorescence than the PBS and FIB groups (Fig. 3A). Quantification of CD86⁺ and CD206⁺ cells per unit area showed that CD86 expression was higher in the PBS and FIB groups (Fig. 3B), while CD206 expression was elevated in the PGF and PGF^−^ groups (Fig. 3C). The calculated M2/M1 ratio was significantly higher in the PGF and PGF^−^ groups, with no statistically significant differences observed between PBS and FIB or between PGF and PGF^−^ (Fig. 3D).

### H&E and Masson’s staining

**Figure 4.**
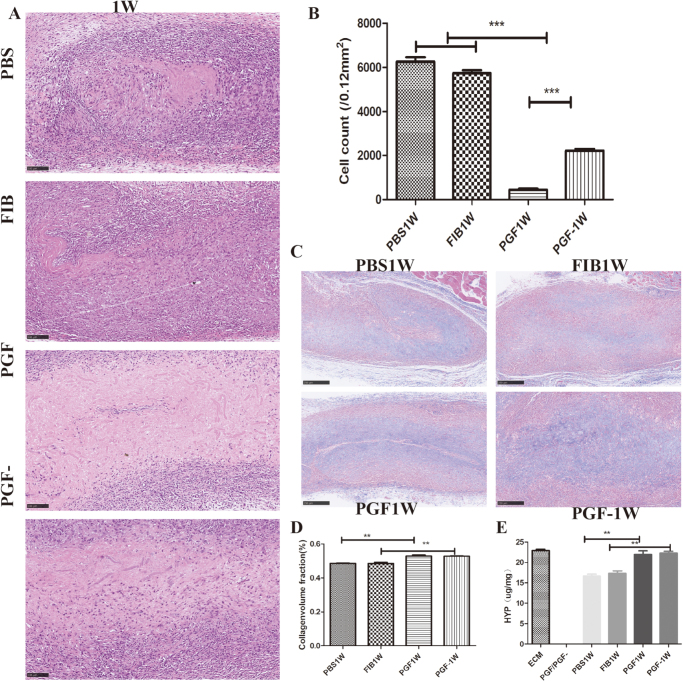
Subcutaneous transplantation, HE staining, and Masson’s staining of TEVGs. (A) HE staining images of TEVGs subcutaneously implanted in rats at week 1; (B) quantification of cell infiltration per unit area at week 1; (C) Masson’s trichrome staining images of TEVGs at week 1 post-implantation; (D) percentage of collagen-stained area per unit area based on Masson’s staining at week 1; and (E) hydroxyproline assay results at different time points post-subcutaneous transplantation. Scale bar: 100 μm. **P < 0.01, ***P < 0.001.

Materials from the different experimental groups were implanted subcutaneously in rats and retrieved for HE staining after 1 week. (The most notable effects were observed at this time point; therefore, only results from the first week are presented.) HE staining was used to assess cell infiltration within the transplanted materials. In the first week, relatively high levels of cell infiltration were observed in both the PBS and FIB groups, whereas the central regions of the PGF and PGF^−^ groups exhibited substantial areas lacking cell infiltration (Fig. 4A). Quantitative analysis of cell counts showed that the PGF group had the lowest number of infiltrating cells, with statistically significant differences compared to the other three groups. No significant difference in cell numbers was observed between the PBS and FIB groups (Fig. 4B).

Masson’s staining was used to evaluate changes in collagen content within the materials after subcutaneous transplantation, thereby providing insight into the *in vivo* degradation rate of ECM. Visual examination of the stained sections indicated that the blue-stained areas in the PGF and PGF^−^ groups during the first week were markedly larger than those in the PBS and FIB groups (Fig. 4C, D). Hydroxyproline assay data from the subcutaneous transplantation experiment further demonstrated that, from the first to the third week, the collagen content in the PGF and PGF^−^ groups remained higher than that in the PBS group. Although the FIB group exhibited slightly greater collagen content than the PBS group, this difference was not statistically significant. Compared to non-transplanted TEVG, the decrease in collagen content observed in the PGF and PGF^−^ groups was less pronounced during the first week (Fig. 4E).

### M1 and M2 cell immunofluorescence staining

**Figure 5.**
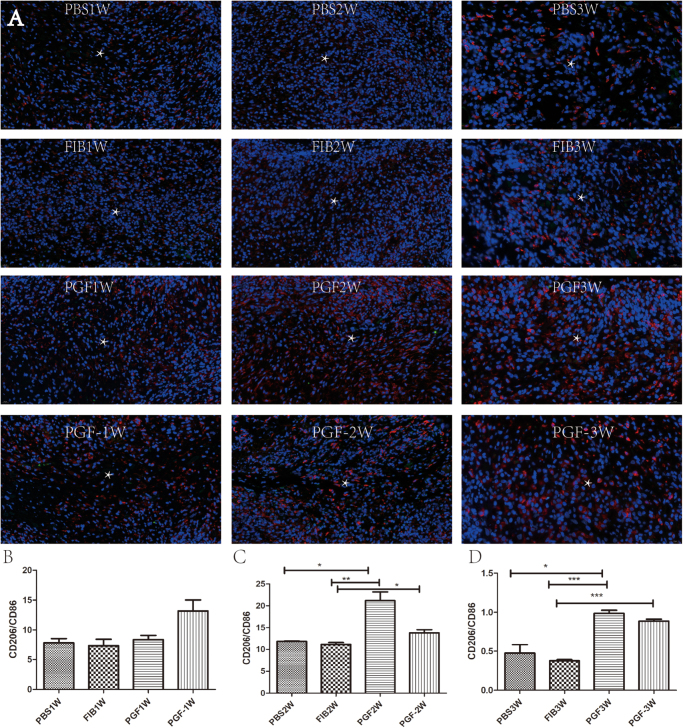
Immunofluorescence staining of TEVGs following subcutaneous transplantation. (A) Representative immunofluorescence images showing CD206 (red), CD86 (green), and DAPI (blue); (B) quantitative analysis of the CD206/CD86 ratio at week 1; (C) CD206/CD86 ratio at week 2; and (D) CD206/CD86 ratio at week 3. Scale bar: 100 μm. *P < 0.05, **P < 0.01, ***P < 0.001.

The asterisk (*) indicates the transplant area. Tissue immunofluorescence staining results following subcutaneous implantation of the materials revealed moderate infiltration of CD206^+^ cells across all four experimental groups from week 1 to week 3, accompanied by a lower abundance of CD86^+^ cells (Fig. 5A). Among these groups, the PBS and FIB groups exhibited a higher number of CD86^+^ cells. Analysis of the CD206/CD86 ratio at each time point indicated no statistically significant difference between groups in the first week (Fig. 5B). However, in weeks 2 and 3, both PGF and PGF^−^ groups displayed significantly higher CD206/CD86 ratios compared to the PBS and FIB groups (Fig. 5C, D). No statistically significant differences were observed between PBS and FIB, or between PGF and PGF^−^.

### Transcriptome sequencing analysis and overview of experimental workflow

**Figure 6.**
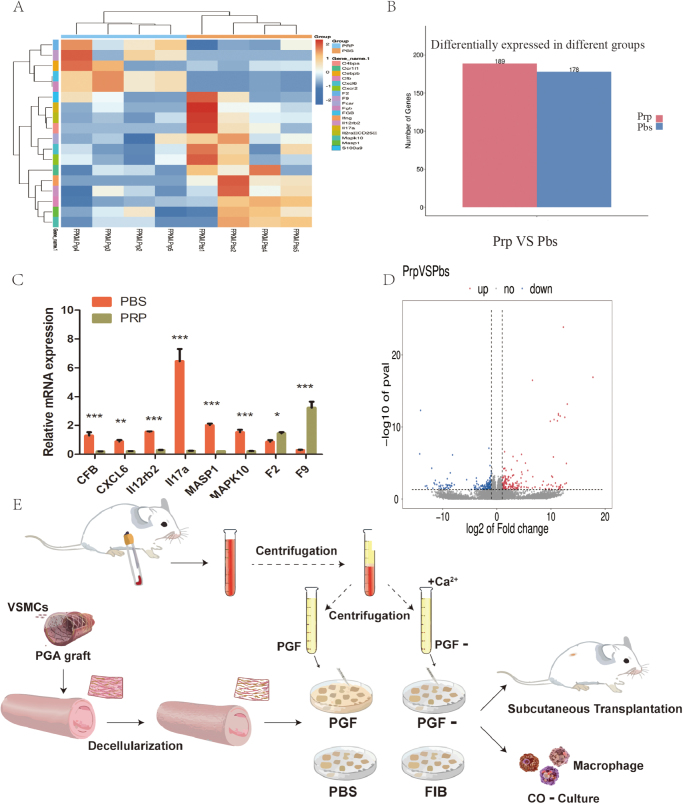
Transcriptomic analysis and experimental workflow. (A) Bar chart showing the number of upregulated and downregulated genes following transcriptome sequencing; (B) Volcano plot illustrating differential gene expression patterns; (C) Kyoto Encyclopedia of Genes and Genomes pathway analysis of differentially expressed genes; (D) RT-qPCR of selected differentially expressed genes ; and (E) Schematic of the experimental procedure. *P < 0.05, **P < 0.01, ***P < 0.001.

Transcriptome sequencing analysis was conducted on subcutaneously implanted TEVG samples in rats after 1 week. The results revealed 189 upregulated and 178 downregulated genes (Fig. 6A, B). Analysis of the top 100 differentially expressed genes indicated that PRP treatment downregulated several inflammation-related genes, including CFB, FGB, and IL17a, which are primarily involved in modulating inflammatory responses and macrophage phagocytic activity. Among the upregulated genes, coagulation-associated genes such as *F2* and *F9* were prominently elevated (Fig. 6C). Selected differentially expressed genes were further validated using qPCR (Fig. 6D).

In this study, whole blood was collected from rat hearts, and PRP was prepared using a two-step low-speed centrifugation protocol. The PRP underwent repeated freeze–thaw cycles, followed by centrifugation to remove the sediment containing red blood cells, white blood cells, and platelets, yielding a PRP solution referred to as PGF. To activate PRP coagulation via calcium ion stimulation, PGF was mixed with 10% calcium gluconate solution, converting soluble fibrinogen into insoluble FIB. The PRP solution lacking fibrinogen – obtained via subsequent centrifugation – was designated as PGF^−^. Finally, TEVGs were mixed with the various treatment groups and transplanted subcutaneously into rats for *in vivo* experimentation (Fig. 6E).

## Discussion

Small-diameter blood vessels (diameter < 6 mm) are mainly used in clinical settings for coronary artery bypass grafting, peripheral arterial reconstruction, and hemodialysis access^[28]^. However, they remain a major challenge in tissue engineering and vascular surgery due to their high susceptibility to thrombosis, intimal hyperplasia, and low long-term patency rates. Autologous vascular grafts (such as the great saphenous vein or radial artery) are considered the gold standard for transplantation, but their availability is limited and harvesting causes additional trauma. Synthetic materials such as ePTFE are suitable for large-diameter vessels but often fail in small-diameter applications because of hemodynamic mismatch and poor biocompatibility^[29]^. In recent years, TEVGs, constructed through the combination of seed cells, biodegradable scaffolds, and growth factors, have shown promising potential, with some products already entering clinical trials. Importantly, the functional mechanism of TEVGs *in vivo* relies on scaffold degradation accompanied by the deposition and remodeling of autologous ECM^[30]^. Therefore, achieving an appropriate degradation profile with minimal inflammatory response is crucial to ensure the efficiency and long-term success of TEVG implantation. Previous studies have reported that M2 macrophages typically display an elongated morphology and are pivotal in facilitating tissue repair while exerting anti-inflammatory functions during the later phases of cellular activity. They serve as key regulators in the resolution of inflammation, largely by releasing anti-inflammatory cytokines such as IL-10 and TGF-β. These cytokines act to suppress excessive immune responses, thereby reducing tissue injury and limiting the progression of inflammatory disorders^[31]^. Although PRP has been widely utilized in clinical practice, its application in TEBVs remains limited. PRP can be autologously obtained and applied, but it remains unclear whether it can modify vascular graft materials to attenuate *in vivo* inflammatory responses and extend material degradation duration. To address this question, TEBV generated via *in vitro* culture was decellularized to remove cellular components. The resulting scaffold material was freeze-dried and subjected to treatment by different experimental groups. Growth factor assays of the culture medium revealed that this composite material was capable of sustained release of PDGF-BB and VEGF for up to 15 days. PRP is known to be enriched with a variety of growth factors^[32]^, among which PDGF-BB and VEGF have been well documented to promote cellular proliferation^[33,34]^.

We evaluated the cytotoxicity and proliferative activity of decellularized vascular graft materials on SMCs by designing distinct experimental groups. Comparison of OD_450_ values on day 3 revealed that ECM concentrations ranging from 50 to 250 ng/mL promoted SMC proliferation. Notably, at 200 ng/mL, TEVG demonstrated a statistically significant enhancement in SMC proliferation; thus, this concentration was selected for subsequent co-culture experiments with macrophages. Macrophages are among the earliest immune cells recruited to the graft site during immune responses and play a pivotal role in graft degradation and tissue regeneration^[35]^. The TEVG materials derived through the aforementioned decellularization method consist predominantly of ECM proteins, primarily collagen, which are essential in promoting host-mediated tissue reconstruction. This regenerative process is intricately linked to macrophage phenotype. Previous studies have shown that decellularized ECM promotes M2 macrophage polarization *in vivo*^[36]^. However, the combined effect of PRP and ECM on macrophage M2/M1 polarization, both *in vivo* and *in vitro*, has not been well characterized. In our processing, we observed an increase in the water absorption capacity of ECM materials treated with FIB, PGF, and PGF^−^, which is presumed to result from interactions among multiple proteins^[37]^. *In vitro* analyses demonstrated that the PBS group alone exhibited marked M1 polarization, likely due to the presence of residual non-degraded PGA in the TEVG. In contrast, both the PGF and PGF^−^ groups significantly promoted M2 polarization, consistent with previous findings on the effect of PRP alone on macrophage phenotype^[38]^.

Yang *et al* fabricated small-diameter vascular graft materials using electrospinning technology; however, the pores of the resulting scaffolds were too small to adequately mimic the native vascular ECM. In contrast, TEVGs form a tubular structure through cell-secreted ECM, which more closely replicates the structural characteristics of native vessels^[39]^. Collagen and elastin are the principal components of ECM^[40]^. To evaluate the effect of PRP incorporation on *in vivo* ECM degradation, various experimental groups were subcutaneously implanted into rats. Collagen content was quantified at different time points using Masson’s trichrome staining and hydroxyproline assay. The results demonstrated that collagen levels in the experimental groups were consistently higher than those in the control group during weeks 1–3. HE staining indicated that, in the first week, cell infiltration in the transplantation area was greater in the PBS and FIB groups compared to the PGF and PGF^−^ groups containing PRP. Immunofluorescence analysis of macrophages revealed a higher M2/M1 ratio in the experimental groups than in the control group. These findings suggest that PRP reduces the inflammatory response to vascular scaffold materials and promotes M2 polarization, thereby decreasing the metabolic rate of ECM in rats. Although fibrinogen is known to influence ECM degradation through multiple mechanisms – including the regulation of ECM structural integrity, modulation of degradative enzyme activity, participation in signal transduction pathways, and involvement in inflammation and tissue repair^[41]^ – this study observed no significant effect of FIB addition on TEVG degradation or inflammatory response. This may be attributed to the limited amount of fibrinogen retained on the TEVG surface. Scanning electron microscopy indicated that while fibrinogen was distributed throughout the TEVG layers, its overall abundance was minimal. In contrast, the PGF group exhibited a denser and more continuous protein network than the FIB group.

This study found that the FIB group – designed to simulate the physiological concentration of fibrinogen in rats – did not significantly influence ECM degradation or macrophage polarization. This outcome may be attributed to the limited adsorption of fibrinogen onto the ECM during the *in vitro* simulation. Although electron microscopy revealed the presence of fibrinogen structures distinct from those of the native ECM, their overall abundance was low. These findings suggest that fibrinogen in plasma is not the primary factor responsible for attenuating ECM degradation during PRP application. Nevertheless, the specific components involved in mediating this effect remain unidentified. Analysis of the four TEVG experimental groups indicated that fibrinogen had no notable impact on inflammatory cell infiltration, macrophage phenotype switching, or ECM degradation. *In vitro* macrophage polarization assays demonstrated that the FIB group yielded results comparable to the PBS group. Whether elevating the concentration of fibrinogen can serve as a substrate for phagocytic activity to delay ECM degradation remains uncertain and warrants further investigation.

To further investigate the underlying mechanisms, transcriptome sequencing revealed that, compared to the PBS group, the PRP group exhibited 189 upregulated genes and 178 downregulated genes. Functional analysis of the top 100 differentially expressed genes indicated that the downregulated genes in the PRP group were primarily associated with inflammatory processes. For example, the gene Cfb (complement factor B) encodes a component of the complement system, which contributes to immune responses, inflammatory regulation, and pathogen clearance. Complement factor B plays a key role in forming the C3 convertase enzyme complex during complement activation, thereby enhancing opsonization and promoting the lysis of pathogens^[42]^. Another downregulated gene, CXCL6 (C-X-C motif chemokine ligand 6), encodes a chemokine involved in inflammation, immune modulation, and tissue repair. CXCL6 acts as a chemoattractant for neutrophils and monocytes, guiding their migration to inflammatory sites and participating in the immune response^[43,44]^. Additionally, several other downregulated genes – Il12rb2 (IL-12 receptor subunit beta 2), Il17a (IL-17A), MASP1 (mannan-binding lectin serine peptidase 1), and MAPK10 (mitogen-activated protein kinase 10) – are closely linked to inflammatory pathways and phagocytic cell activation^[43,45–47]^. Conversely, among the upregulated genes, F2 (coagulation factor II) and F9 (coagulation factor IX) were notable, both of which are primarily involved in coagulation cascades and may contribute to cell proliferation and differentiation^[48]^. Overall, transcriptomic findings suggest that PRP treatment suppresses genes related to inflammation and immune cell chemotaxis while enhancing genes related to coagulation. These results imply that, although PRP may reduce inflammation, its procoagulant properties warrant careful consideration in future vascular graft applications.

This study has several limitations. First, the differentially expressed genes identified through transcriptome sequencing have not yet undergone functional validation, and the key signaling pathways involved remain unidentified. Second, the observation period for TEVG implantation was relatively short, and the material has not been tested in *in situ* vascular transplantation models. Third, it is still unclear whether PRP application may elicit adverse immune responses. These issues will be addressed in future investigations.

## Conclusion

Combining PRP with decellularized TEVG materials creates a more protective microenvironment that enhances the water absorption capacity of the graft, promotes a shift toward reparative macrophage phenotypes, reduces early inflammatory cell infiltration, and slows the degradation of ECM components *in vivo*. Results from rat subcutaneous implantation models and transcriptomic analysis demonstrate that PRP treatment decreases the number of M1 macrophages while increasing the number of M2 macrophages, thereby elevating the M2/M1 ratio and promoting a reparative immune microenvironment. Moreover, PRP downregulates the expression of multiple inflammation-associated genes, mitigating immune activation and improving the biocompatibility of the implanted material. These findings provide a promising direction for future applications in *in situ* vascular transplantation. Although PRP also upregulates genes associated with coagulation, this effect may be attributed to the presence of fibrinogen and specific PRP-derived growth factors. In conclusion, PRP enhances the biocompatibility of TEVG materials and offers valuable insight for the clinical application of TEVGs.

## Data Availability

The datasets generated and/or analyzed during the current study are available from the corresponding author on reasonable request.

## References

[1] Lawson JH Glickman MH Ilzecki M. Bioengineered human acellular vessels for dialysis access in patients with end-stage renal disease: two phase 2 single-arm trials. Lancet 2016;387:2026–34. 27203778 10.1016/S0140-6736(16)00557-2 PMC4915925

[2] Ding K Yu X Wang D. small diameter expanded polytetrafluoroethylene vascular graft with differentiated inner and outer biomacromolecules for collaborative endothelialization, anti-thrombogenicity and anti-inflammation. Colloids Surf B Biointerfaces 2023;229:113449. 37506438 10.1016/j.colsurfb.2023.113449

[3] Scheiner KC Maas-Bakker RF Nguyen TT. Sustained release of vascular endothelial growth factor from poly(epsilon-caprolactone-PEG-epsilon-caprolactone)-b-Poly(l-lactide) multiblock copolymer microspheres. ACS Omega 2019;4:11481–92. 31460253 10.1021/acsomega.9b01272 PMC6681988

[4] Skovrind I Harvald EB Juul BH. Concise review: patency of small-diameter tissue-engineered vascular grafts: a meta-analysis of preclinical trials4. Stem Cells Transl Med 2019;8:671–80. 30920771 10.1002/sctm.18-0287 PMC6591545

[5] Tresoldi C Pellegata AF Mantero S. Cells and stimuli in small-caliber blood vessel tissue engineering4. Regener Med 2015;10:505–27. 10.2217/rme.15.19 26022767

[6] Liu J Gao Y Wang S. Effect of operation-synchronizing transfusion of apoptotic spleen cells from donor rats on acute rejection of recipient rats after liver transplantation. World J Gastroenterol 2005;11:1161–66. 15754397 10.3748/wjg.v11.i8.1161 PMC4250706

[7] Hlapcic I Belamaric D Bosnar M. Combination of systemic inflammatory biomarkers in assessment of chronic obstructive pulmonary disease: diagnostic performance and identification of networks and clusters. Diagnostics (Basel) 2020;10:1029. 33266187 10.3390/diagnostics10121029 PMC7760570

[8] Chen YT Lee HS Hsieh DJ. 3D composite engineered using supercritical CO (2) decellularized porcine cartilage scaffold, chondrocytes, and PRP: role in articular cartilage regeneration. J Tissue Eng Regen Med 2021;15:163–75. 33258246 10.1002/term.3162

[9] Su K Bai Y Wang J. Comparison of hyaluronic acid and PRP intra-articular injection with combined intra-articular and intraosseous PRP injections to treat patients with knee osteoarthritis. Clin Rheumatol 2018;37:1341–50. 29388085 10.1007/s10067-018-3985-6

[10] McLeod MD Austen WJ. Commentary on: effect of use of platelet-rich plasma (PRP) in skin with intrinsic aging process. Aesthet Surg J 2018;38:329–31. 29126293 10.1093/asj/sjx193

[11] Baklaushev VP Bogush VG Kalsin VA. Tissue engineered neural constructs composed of neural precursor cells, recombinant spidroin and PRP for neural tissue regeneration. Sci Rep 2019;9:3161. 30816182 10.1038/s41598-019-39341-9 PMC6395623

[12] Wu YD Jiang HJ Zhou HH. PRP significantly promotes the adhesion and migration of vascular smooth muscle cells on stent material. Eur J Med Res 2023;28:581. 38071348 10.1186/s40001-023-01541-5 PMC10710707

[13] Zhan X Gao M Jiang Y. Nanofiber scaffolds facilitate functional regeneration of peripheral nerve injury. Nanomedicine 2013;9:305–15. 22960189 10.1016/j.nano.2012.08.009

[14] Guelcher SA. Biodegradable polyurethanes: synthesis and applications in regenerative medicine. Tissue Eng Part B Rev 2008;14:3–17. 18454631 10.1089/teb.2007.0133

[15] Yilgor P Sousa RA Reis RL. Effect of scaffold architecture and BMP-2/BMP-7 delivery on in vitro bone regeneration. J Mater Sci Mater Med 2010;21:2999–3008. 20740306 10.1007/s10856-010-4150-1

[16] Xu P Wu Y Zhou L. Platelet-rich plasma accelerates skin wound healing by promoting re-epithelialization. Burns Trauma 2020;8:tkaa028. 32821743 10.1093/burnst/tkaa028 PMC7427034

[17] TITAN Group. Agha RA Mathew G Rashid R. Transparency In The reporting of Artificial INtelligence the TITAN guideline. Prem J Sci 2025;10:100082.

[18] Choudhary OP Infant SS Chopra H. Exploring the potential and limitations of artificial intelligence in animal anatomy. Ann Anat 2025;258:152366. 39631569 10.1016/j.aanat.2024.152366

[19] Vickram AS Infant SS Chopra H. AI-powered techniques in anatomical imaging: impacts on veterinary diagnostics and surgery. Ann Anat 2025;258:152355. 39577814 10.1016/j.aanat.2024.152355

[20] Kilkenny C Browne WJ Cuthill IC. Improving bioscience research reporting: the ARRIVE guidelines for reporting animal research. PLoS Biol 2010;8:e1000412. 20613859 10.1371/journal.pbio.1000412 PMC2893951

[21] Dziki JL Huleihel L Scarritt ME. Extracellular matrix bioscaffolds as immunomodulatory biomaterials. Tissue Eng Part A 2017;23:1152–59. 28457179 10.1089/ten.tea.2016.0538 PMC6112165

[22] Huber SC de Lima MS Sachetto Z. Characterization of autologous platelet rich plasma (PRP) and its biological effects in patients with Behcet’s Disease. Regen Ther 2021;18:339–46. 34584910 10.1016/j.reth.2021.08.010 PMC8441104

[23] Dankovich TM Kaushik R Olsthoorn L. Extracellular matrix remodeling through endocytosis and resurfacing of Tenascin-R. Nat Commun 2021;12:7129. 34880248 10.1038/s41467-021-27462-7 PMC8654841

[24] Pustlauk W Westhoff TH Claeys L. Induced osteogenic differentiation of human smooth muscle cells as a model of vascular calcification. Sci Rep 2020;10:5951. 32249802 10.1038/s41598-020-62568-w PMC7136202

[25] Barrett JP Costello DA O’Sullivan J. Bone marrow-derived macrophages from aged rats are more responsive to inflammatory stimuli. J Neuroinflammation 2015;12:67. 25890218 10.1186/s12974-015-0287-7 PMC4397943

[26] Cissell DD Link JM Hu JC Athanasiou KA. A modified hydroxyproline assay based on hydrochloric acid in Ehrlich’s solution accurately measures tissue collagen content. Tissue Eng Part C Methods 2017;23:243–50. 28406755 10.1089/ten.tec.2017.0018 PMC5397204

[27] Choudhary OP Choudhary P. Scanning electron microscope: advantages and disadvantages in imaging components. Int J Curr Microbiol Appl Sci 2017;6:1877–82.

[28] Lawson JH Niklason LE Roy-Chaudhury P. Challenges and novel therapies for vascular access in haemodialysis. Nat Rev Nephrol 2020;16:586–602. 32839580 10.1038/s41581-020-0333-2 PMC8108319

[29] Hu YT Pan XD Zheng J. In vitro and in vivo evaluation of a small-caliber coaxial electrospun vascular graft loaded with heparin and VEGF. Int J Surg 2017;44:244–49. 28648794 10.1016/j.ijsu.2017.06.077

[30] Niklason LE Lawson JH. Bioengineered human blood vessels. Science 2020;370:eaaw8682. 33033191 10.1126/science.aaw8682

[31] Murray PJ Allen JE Biswas SK. Macrophage activation and polarization: nomenclature and experimental guidelines. Immunity 2014;41:14–20. 25035950 10.1016/j.immuni.2014.06.008 PMC4123412

[32] Hamilton B Tol JL Knez W. Exercise and the platelet activator calcium chloride both influence the growth factor content of platelet-rich plasma (PRP): overlooked biochemical factors that could influence PRP treatment. Br J Sports Med 2015;49:957–60. 23770705 10.1136/bjsports-2012-091916

[33] Hong HK Park YJ Kim DK. Preclinical efficacy and safety of VEGF-Grab, a novel Anti-VEGF drug, and its comparison to aflibercept. Invest Ophthalmol Vis Sci 2020;61:22. 10.1167/iovs.61.13.22 PMC7671872 33196778

[34] Zhao FY Xu SL Zhang CF. PDGF mediates pulmonary arterial smooth muscle cell proliferation and migration by regulating NFATc2. Mol Med Rep 2021;23:39. 33179105 10.3892/mmr.2020.11677 PMC7684858

[35] Mirmalek-Sani SH Sullivan DC Zimmerman C. Immunogenicity of decellularized porcine liver for bioengineered hepatic tissue. Am J Pathol 2013;183:558–65. 23747949 10.1016/j.ajpath.2013.05.002 PMC3730770

[36] Qiu X Liu S Zhang H. Mesenchymal stem cells and extracellular matrix scaffold promote muscle regeneration by synergistically regulating macrophage polarization toward the M2 phenotype. Stem Cell Res Ther 2018;9:88. 29615126 10.1186/s13287-018-0821-5 PMC5883419

[37] Alvarez-Castillo E Bengoechea C Guerrero A. Strengthening of porcine plasma protein superabsorbent materials through a solubilization-freeze-drying process. Polymers (Basel) 2021;13:772. 33802290 10.3390/polym13050772 PMC7959129

[38] Uchiyama R Toyoda E Maehara M. Effect of platelet-rich plasma on M1/M2 macrophage polarization. Int J Mol Sci 2021;22:2336. 33652994 10.3390/ijms22052336 PMC7956636

[39] Yang Y Lei D Zou H. Hybrid electrospun rapamycin-loaded small-diameter decellularized vascular grafts effectively inhibit intimal hyperplasia. Acta Biomater 2019;97:321–32. 31523025 10.1016/j.actbio.2019.06.037

[40] Rebelo SP Pinto C Martins TR. 3D-3-culture: a tool to unveil macrophage plasticity in the tumor microenvironment. Biomaterials 2018;163:185–97. 29477032 10.1016/j.biomaterials.2018.02.030

[41] Jenkins RG Su X Su G. Ligation of protease-activated receptor 1 enhances alpha(v)beta6 integrin-dependent TGF-beta activation and promotes acute lung injury. J Clin Invest 2006;116:1606–14. 16710477 10.1172/JCI27183 PMC1462943

[42] Thakkinstian A McEvoy M Chakravarthy U. The association between complement component 2/complement factor B polymorphisms and age-related macular degeneration: a HuGE review and meta-analysis. Am J Epidemiol 2012;176:361–72. 22869612 10.1093/aje/kws031 PMC6483268

[43] Maksym RB Tarnowski M Grymula K. The role of stromal-derived factor-1–CXCR7 axis in development and cancer. Eur J Pharmacol 2009;625:31–40. 19835865 10.1016/j.ejphar.2009.04.071 PMC2783873

[44] O’Garra A Redford PS McNab FW. The immune response in tuberculosis. Annu Rev Immunol 2013;31:475–527. 23516984 10.1146/annurev-immunol-032712-095939

[45] Coffey ET. Nuclear and cytosolic JNK signalling in neurons. Nat Rev Neurosci 2014;15:285–99. 24739785 10.1038/nrn3729

[46] Weisel JW. Structure of fibrin: impact on clot stability. J Thromb Haemost 2007;5:116–24. 17635717 10.1111/j.1538-7836.2007.02504.x

[47] Ariens RA. Fibrin(ogen) and thrombotic disease. J Thromb Haemost 2013;11:294–305. 23809133 10.1111/jth.12229

[48] Nerlov C. The C/EBP family of transcription factors: a paradigm for interaction between gene expression and proliferation control. Trends Cell Biol 2007;17:318–24. 17658261 10.1016/j.tcb.2007.07.004

